# Osteoporosis is associated with varus deformity in postmenopausal women with knee osteoarthritis: a cross-sectional study

**DOI:** 10.1186/s12891-021-04580-3

**Published:** 2021-08-14

**Authors:** Cheng Zhang, Zhikun Zhuang, Xiaojun Chen, Keda Li, Tianye Lin, Fengxiang Pang, Ying Zhang, Wei He, Qiushi Wei

**Affiliations:** 1 The First Clinical Medical College of Guangzhou University of Chinese Medicine, 510405 Guangzhou, Guangdong China; 2 Quanzhou Osteopathic Hospital, Fujian University of Traditional Chinese Medicine, Fujian 362000 Quanzhou, China; 3 Liaoning University of Chinese Medicine, 110033 Shengyang, Liaoning China; 4 Henan Provincial Orthopedic Hospital, Henan 471000 Luoyang, China; 5 Department of Joint Orthopaedic, the Third Affiliated Hospital, Guangzhou University of Chinese Medicine, 510405 Guangzhou, Guangdong China; 6 Institude of Orthopedica, Guangzhou University of Chinese Medicine, 510405 Guangzhou, Guangdong China

**Keywords:** Knee osteoarthritis, Varus deformity, Bone mineral density, Limb alignment Osteoporosis

## Abstract

**Background:**

Varus deformity of the knee is a common pathological characteristic in knee osteoarthritis (KOA), and not enough attention has been given to the relationship between knee varus deformity and the state of systemic bone mass. The purpose of this study was to evaluate the potential relationship between bone mineral density (BMD) and varus deformity in postmenopausal women with KOA.

**Methods:**

A total of 202 postmenopausal women with KOA(KL grade ≥ 2)in our department from January 2018 to June 2020 were reviewed in this cross-sectional study. The hip-knee-ankle angle of the lower extremity (HKA), medial distal femoral angle (MDFA), medial proximal tibial angle (MPTA), and the angle of the joint line (JLCA) were measured in all patients. According to the HKA Angle, these participants were divided into the varus deformity group (HKA < 175.3°) and the normal limb alignment group (175.3°≤ HKA ≤ 180.3°). The BMD of the lumbar (L1-L4), left femoral neck, and left hip were measured by dual-energy X-ray absorptiometry in all patients. The difference in BMD between the knee varus deformity group and the normal limb alignment group was compared, and the relationship between the different angles of limb alignment and the BMD values at different sites was evaluated.

**Results:**

There were 144 cases (71.3 %) in the varus deformity group and 58 cases (28.7 %) in the normal limb alignment group. BMD at different joint sites within the knee varus deformity group was lower than of the normal limb alignment group, and the prevalence of osteoporosis was higher. After adjusting for confounding factors such as age, BMI, pain duration, and affected side, binary logistic regression showed that osteoporosis was an independent risk factor for varus deformity of KOA, and multiple linear regression showed that the BMD of spine, femoral neck, and hip was significantly associated with varus deformity of KOA. Pearson correlation analysis showed that BMD of the lumbar spine (L1-L4), left femoral neck and left hip joint were positively correlated with the HKA, but negatively correlated with JLCA. MPTA was positively correlated with the left femoral neck and left hip joint BMD, but not correlated with lumbar bone density. Furthermore, in the normal limb alignment group, the HKA was only negatively correlated with JLCA, but not significantly correlated with MDFA and MPTA. In the varus deformity group, the HKA was not only negatively correlated with JLCA but also positively correlated with MDFA and MPTA.

**Conclusions:**

Osteoporosis should be a major risk factor for varus deformity in postmenopausal women with KOA. The progression of varus deformity of the knee should be concerned in postmenopausal women who simultaneously has KOA and osteoporosis.

## Background

Knee osteoarthritis (KOA) and osteoporosis are two common diseases in orthopedics. Because the two diseases share common pathogenic factors, such as age, gender, heredity, previous trauma and inflammation [1, 2], they often coexist in the same patient, especially middle-aged and elderly postmenopausal women [1, 3]. Furthermore, the two diseases are also the main diseases affecting the quality of life of middle-aged and elderly people [4, 5]. Experiencing long-term pain and limited activity lead to a decline in the labor force, which imposes an additional economic burden on society [6].

KOA is widely considered a disease of joint articular cartilage with subchondral bone changes and localised inflammation [7]. Among these effects, increasing attention has been paid to the role of subchondral bone changes in the pathological process of KOA. In previous studies, researchers only focused on the sclerosis of the subchondral plate and the formation of surrounding osteophytes in patients with advanced KOA, suggesting that KOA is associated with high bone density [8, 9]. However, in recent years, more studies have found that in the early stage of KOA, subchondral bone resorption increases and bone strength decreases, which in turn cause degeneration of the cartilage [10, 11]. Nonetheless, when osteoporosis and KOA coexist in the same patient, there is debate about whether systemic low bone mass exacerbates the progression of KOA [12, 13].

Varus deformity of the knee joint is not only a key node in the pathological process of KOA but also an important factor for the rapid degeneration of the knee joint. Therefore, the prevention of varus deformity can greatly delay the speed of joint degeneration, which is the key to prevent and treat severe KOA. It is well known that varus deformity may result from congenital anatomical abnormalities, or it may be secondary to medial articular space narrowing after cartilage wear and subluxation of the tibiofemoral joint [14]. However, this cannot fully account for all clinical varus deformity. Although much attention has been given to the effect of periarticular bone mineral density (BMD) on limb alignment [15, 16], the effect of systemic BMD, which represents the level of general bone mass, on limb alignment has not been adequately studied.

The purpose of this study was to investigate the relationship between the different angles of limb alignment and the general bone mass in postmenopausal women with KOA, with or without varus alignment. Our hypothesis was that systemic low bone mass may be an important risk factor for varus deformity in postmenopausal women with KOA.

## Methods

We included postmenopausal women hospitalized in our orthopedic department with a diagnosis of knee osteoarthritis from January 2018 to June 2020. All of these patients met the American College of Rheumatology diagnostic criteria for KOA [17]. After admission, full-length weighted X-rays of bilateral lower extremities were taken for all patients, and BMD of the lumbar spine (LI-L4), left femoral neck and left hip were measured by dual-energy X-ray absorptiometry. The Kellgren-Lawrence (KL) grade was evaluated by a bilateral lower extremity X-ray [18]. Clinical data, such as age, duration of pain, menstrual history, trauma history, and recent medication use were collected using an inpatient electronic medical record system. Height and weight were measured prior to the bone density test, and body mass index (BMI) was calculated using height and weight. In order to reduce the bias, the following exclusion criteria were established: first, patients with knee valgus deformity, as knee valgus is relatively rare clinically and the number of cases is relatively small [19]; second, patients who had taken drugs affecting bone metabolism, such as bisphosphonates and hormones, in the last half year; third, a full-length X-ray of both lower limbs in nonstandard anteroposterior standing position to exclude the measurement of limb alignment angles caused by rotation of lower extremities; fourth, patients with extra-articular malformations that may affect the distribution of the force lines of the knee, such as severe scoliosis, pelvic tilt, hip dysplasia, and angular malformation of the femoral shaft or tibial shaft, etc.; fifth, patients with pain so severe they cannot walk to reduce the effects of disuse osteoporosis.

### Measurement of different angles of limb alignment

**Fig. 1:**
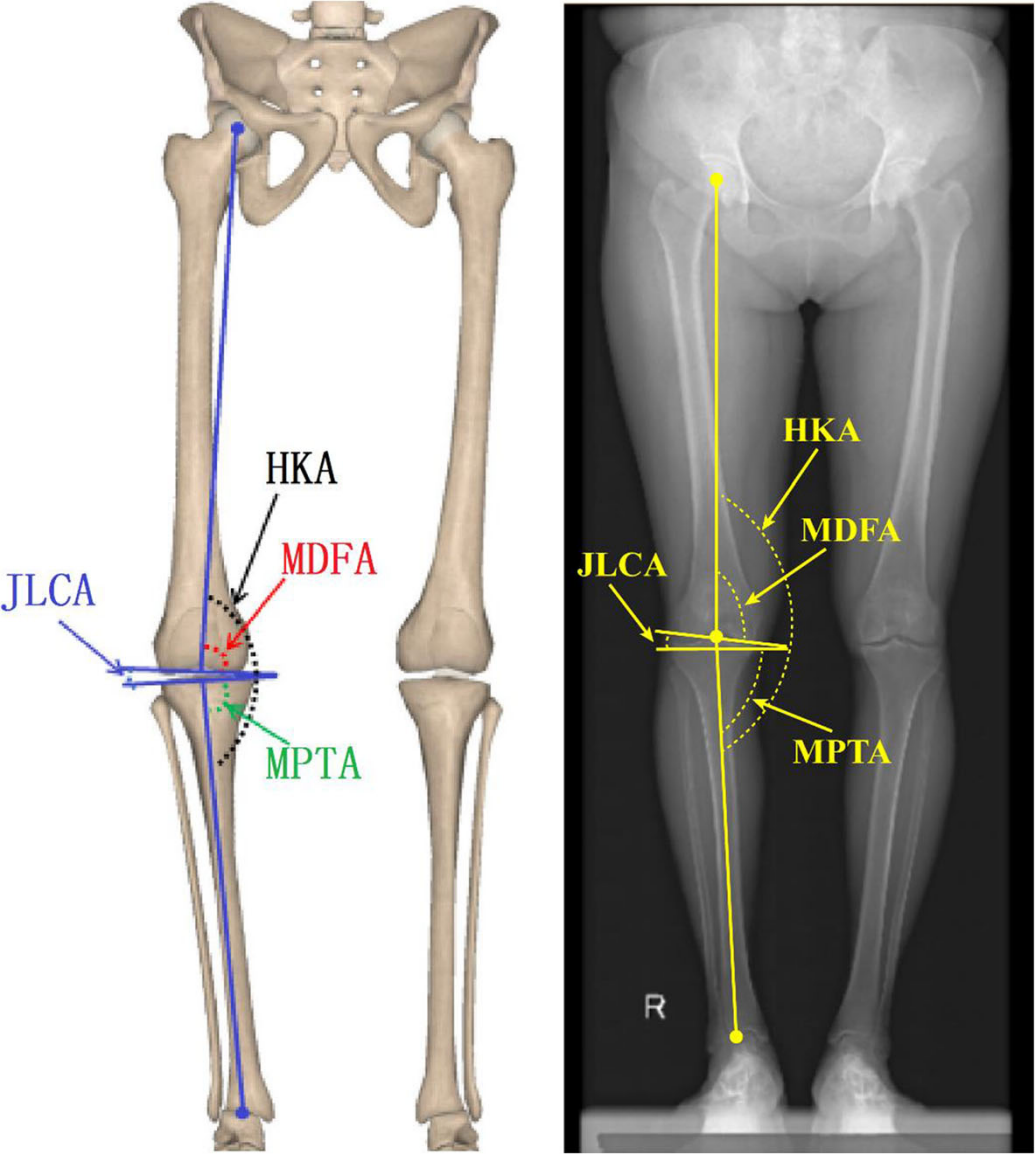
Schematic diagram of mechanical angle of lower extremity (HKA), distal medial femoral angle (MDFA), proximal medial tibial angle (MPTA) and angle of joint line (JLCA)

After admission, full-length weight-bearing X-ray films of both lower limbs were taken. And four different angles, hip-knee-ankle angle (HKA), distal femoral medial angle (MDFA), proximal tibial medial angle (MPTA) and the angle of the joint line (JLCA), were measured. If patients had been diagnosed with bilateral KOA, the more severe side was measured (The severity was determined by the KL score. If both knees had the same KL scores, the side with the smaller HKA was defined as the more severe). The mechanical axis of the femur was defined as the connection between the center of the femoral head and the center of the femoral condyle, while the mechanical axis of the tibia was defined as the connection between the center of the tibial plateau and the center of the ankle joint [20]. The degree of knee varus was defined by the HKA, i.e., the medial intersection angle between the mechanical axis of the femur and the mechanical axis of the tibia [20]. The smaller the HKA, the more serious the varus deformity. According to the standard range of the physiological HKA of Chinese women, we defined 175.3°≤ HKA ≤ 180.3° as normal limb alignment and an HKA < 175.3° as varus deformity [21]. The other three angles were defined as follows: MDFA as the medial angle between the mechanical shaft of the femur and the tangential line of the femoral condyle, MPTA as the medial angle between the mechanical shaft of the tibia and the tangential line of the tibial plateau, and JLCA as the lateral angle between the tibial plateau tangent and the femoral condyle tangent (Fig. 1).

To reduce measurement errors, the same researcher (C Zhang) took all measurements using the same imaging system successively at 2-week intervals. If the error between two measurements of either angle is greater than 0.5°, another researcher (QS Wei) will make the measurement .The investigator was unaware of the patient’s bone mineral density and other clinical data prior to measurement.

### Measurement of bone density

After admission, BMD was measured at the left femoral neck, left hip and lumbar spine (L1-L4) by dual energy X-ray absorptiometry (GE Lunar Prodigy, USA). The BMD of these three regions can sufficiently reflect the bone mass of the whole body and is the gold standard for the diagnosis of osteoporosis [22]. According to WHO diagnostic criteria, T≤-2.5 is diagnosed as osteoporosis, -2.5 < T<-1 is diagnosed as osteopenia, and ≥-1 is normal bone density [23]. If the T-value of any of the three sites (spine, hip or femoral neck) met the diagnostic criteria for osteoporosis, the patient was diagnosed with osteoporosis.

### Statistical analysis

After the normal distribution test of the numerical variables, the Student ‘s t-test was used for the intergroup comparison of age, BMI and BMD values at different sites. Conversely, the Mann-Whitney U test was used for the intergroup comparison of the duration of pain. The chi-square test was used to compare the prevalence of osteoporosis. Binary logistic regression analysis was used to determine the risk factors affecting varus deformity. The Pearson correlation coefficient was used to evaluate the correlation between BMD at different positions and different angles of limb alignment. Multiple linear regression was used to evaluate the influence of BMD values at different sites on the HKA after adjusting for confounding factors, such as age, BMI and duration of pain. Finally, the Pearson correlation coefficient was used to evaluate the correlation between the HKA and other angles in the varus group and the normal limb alignment group.

All data analyses were performed with IBM SPSS 24.0 statistical software, and a *P*-value less than 0.05 was considered statistically significant in all tests.

## Results

Two hundred and two postmenopausal women with KOA were enrolled, with an average age of 65.5 ± 7.4 years (49–83 years), an average BMI of 26.2 ± 3.8 kg/m2, a median pain duration of 60 months (0.5–360 months). Among the 202 cases, there are 44 cases were classified as grade 2, 79 cases as grade 3, 79 cases as grade 4 according to the KL grade classification. Eighteen-four cases were diagnosed with osteoporosis, 94 cases with osteopenia, and the remaining 24 cases were normal. In the varus deformity group (HKA < 175.3°), there were 144 cases, including 69 cases of osteoporosis, 60 cases of osteopenia and 15 cases of normal bone mass. There were 58 cases in the normal limb alignment group (175.3°≤HKA ≤ 180.3°), including 15 cases of osteoporosis, 34 cases of osteopenia, and 9 cases of normal bone mass. There were no statistically significant differences in age, BMI and pain duration between the two groups. The BMD of the lumbar spine (L1-L4), left femoral neck and left hip of the varus deformity group was significantly lower than that of the normal limb alignment group, and the prevalence of osteoporosis (47.9 %) was significantly higher than that of the normal limb alignment group (25.9 %, *P* < 0.05) (Table 1).

**Table 1:** Comparison of characteristics, bone mineral density and prevalence of osteoporosis

	Total	Varus group	Normal alignment group	*p-*value
Numbers	202	144	58	
Age (years)	65.5 ± 7.4	66.1 ± 7.6	64.0 ± 6.7	0.068
BMI (kg/m^2^)	26.2 ± 3.8	26.1 ± 3.9	26.3 ± 3.3	0.808
Pain duration (month)	60 (0.5–360)	60 (0.5–360)	36 (1.0-240.0)	0.355
**BMD (g/cm**^**2**^**)**				
L1-L4 Spine	0.87 ± 0.16	0.84 ± 0.15	0.94 ± 0.17	< 0.001
Femoral neck	0.69 ± 0.14	0.67 ± 0.13	0.74 ± 0.13	0.002
Total hip	0.80 ± 0.14	0.78 ± 0.14	0.85 ± 0.13	0.001
**Prevalence**				0.006
Osteoporosis (N, %)	84(42)	69 (47.9)	15 (25.9)	
Osteopenia (N, %)	94(47)	60 (41.7)	34 (58.6)	
Normal BMD (N, %)	24(11)	15 (10.4)	9 (15.5)	
**Affected side**				0.424
Left (N, %)	113(56 %)	78(54)	35(60)	
Right (N, %)	89(44 %)	66(46)	23(40)	

Data are presented as the mean ± SD for age, BMI,BMD, and median (Least - most) for pain duration

*BMI* body mass index, *BMD* bone mineral density, *SD* standard deviation

Binary logistic regression showed that osteoporosis could significantly influence the occurrence of knee varus deformity. After adjusting for confounding factors, such as age, BMI, pain duration and affected side, the multiple logistic regression revealed osteoporosis was an independent risk factor for varus deformity (*p* = 0.034), while osteopenia was not (*p* = 0.929). The risk of varus deformity was 2.868 times higher in patients with osteoporosis than in those with normal bone mass. In addition, the BMD of the lumbar spine (L1-L4), the left femoral neck, and the left hip were also significantly associated with a lower risk of varus deformity (*p* = 0.001, 0.011, 0.006 respectively) (Table 2).

**Table 2:** The influence of systemic bone mass on the varus deformity

	Crude			Adjusted^a^		
	β	OR[95% CI]	*p-*value	β	OR[95% CI]	*p-*value
Bone mass state						
Osteoporosis	1.015	2.760[1.018-7.483]	0.046	0.862	2.868 [1.035-7.645]	0.034
Osteopenia	0.057	1.059[0.419-2.676]	0.904	-0.046	0.955[[0.349-2.616]	0.929
Normal	/	/	/	/	/	/
BMD						
L1-L4 Spine	-3.693	0.025[0.003-0.184]	<0.001	-3.833	0.022[0.002-0.192]	0.001
Femoral neck	-3.507	0.030[0.003-0.304]	0.003	-3.610	0.027[0.002-0.444]	0.011
Total hip	-3.917	0.020[0.002-0.217]	0.001	-3.985	0.019[0.001-0.322]	0.006

*BMI* body mass index, *OR* odds ratio, *CI* confidence interval

^a^Adjusted for age, BMI, pain duration and affected side

**Fig. 2:**
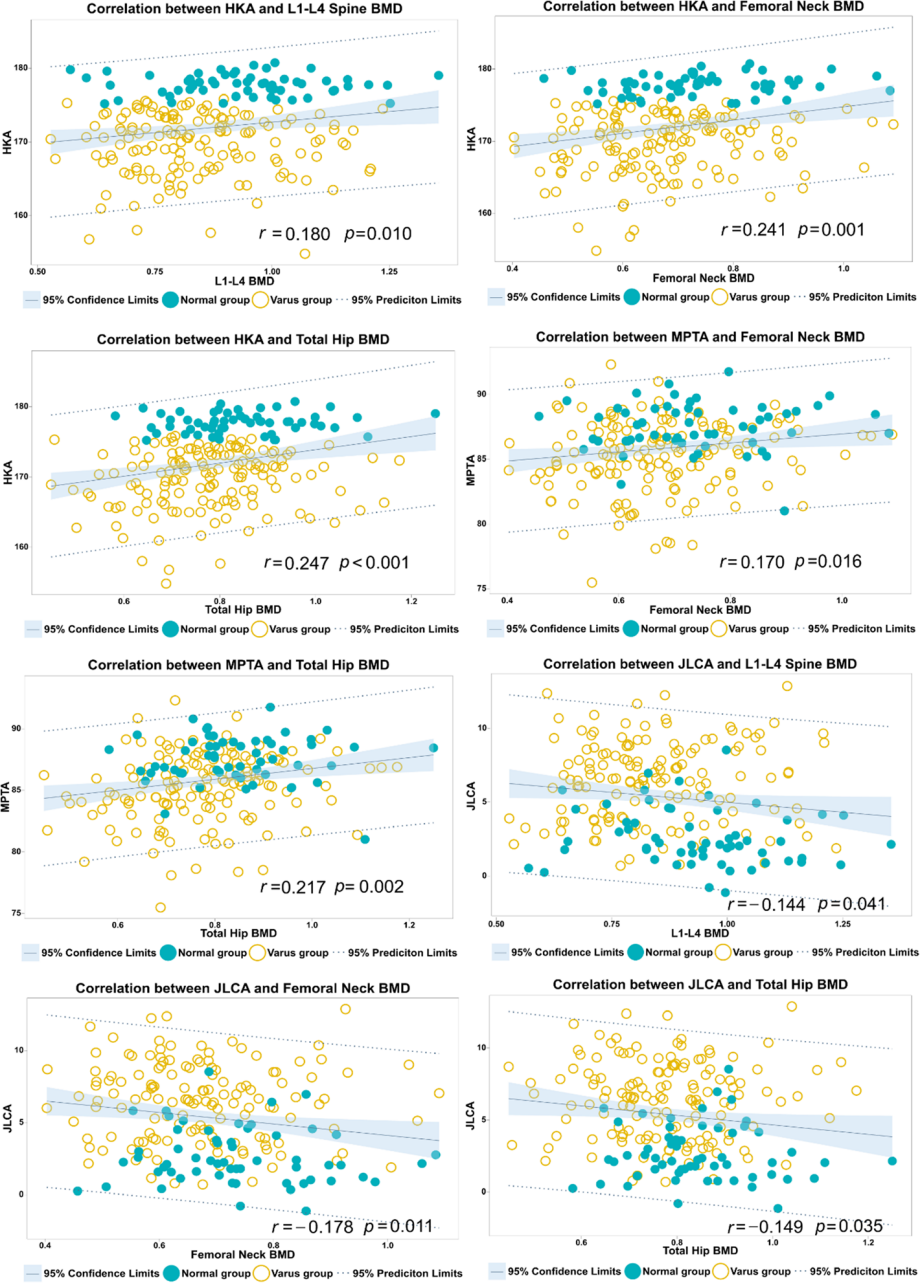
Correlation between BMD values of different sites and different angles of limb alignment. BMD: bone mineral density, HKA: hip-knee-ankle angle, MDFA: distal femoral medial angle, MPTA: proximal tibial medial angle, JLCA: angle of the joint line

According to Pearson correlation analysis, among all participants, the BMD of the lumbar spine (L1 - L4), the left femoral neck and left hip was positively correlated with the HKA, and negatively correlated with JLCA. MPTA was positively correlated with BMD values of the left femoral neck and the left hip joint, but not with those of the lumbar spine. MDFA was not correlated with BMD values of the above three sites (Fig. 2).

In the multiple linear regression model, after adjusting for confounding factors, such as age, BMI, and pain duration, the BMD of the lumbar spine (L1-L4), the left femoral neck, and the left hip have a significant positive influence on the value of the HKA (Table 3). That is, with increasing BMD, the degree of varus deformity would be reduced.

**Table 3:** The relationship between BMD at different sites and HKA after adjusting for confounding factors

	B	S.E.	Standardized β	95 % CI for B	*p-*value
L1-L4 BMD	5.841	2.284	0.183	1.337–10.334	0.011
Femoral neck BMD	8.980	2.963	0.235	3.137–14.823	0.003
Total hip BMD	7.631	2.961	0.202	1.792–13.469	0.011

Adjusted for age, BMI, pain duration and affected side

*HKA* hip-knee-ankle angle, *BMD* bone mineral density, *CI* confidence interval

**Fig. 3:**
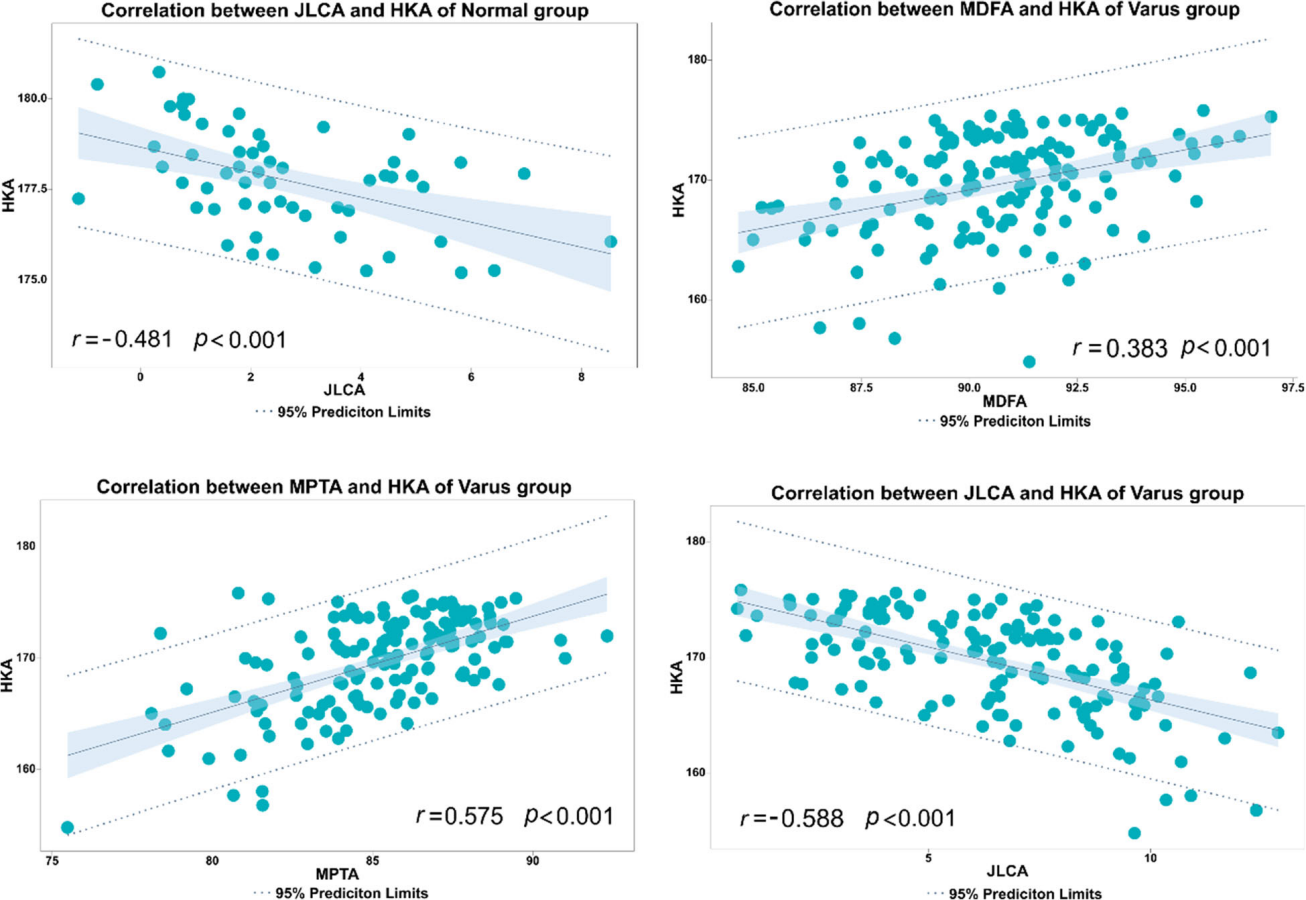
Correlation analysis of the HKA with MDFA, MPTA and JLCA

Finally, in the normal limb alignment group, Pearson correlation analysis showed that the HKA was only negatively correlated with JLCA but had no significant correlation with MDFA and MPTA, whereas in the varus deformity group, HKA was not only negatively correlated with JLCA but also positively correlated with MDFA and MPTA (Fig. 3).

## Discussion

In early studies, high bone mineral density was generally believed to be related to the incidence and progression of osteoarthritis[8, 9]. Although recent studies began to realize that osteoporosis plays an important role in the pathological process of KOA, the relationship between osteoporosis and KOA is still controversial. Part of the reason may be that patients in different studies were at different stages of disease and had different indicators for measuring the progression of KOA [24]. KL grade pays more attention to the formation of osteophyte and the narrowing of joint space [25], while limb alignment can more directly reflect the incidence of varus deformity, which is key to the rapid progression of KOA.

Previous studies have primarily focused on the relationship between the subchondral BMD and limb alignment. Yoshinori et al. found that in patients with medial compartment KOA, the medial condyles had significantly higher BMD than the lateral condyles in both the femur and tibia, and the medial-to-lateral BMD ratio was negatively correlated with the HKA and the inclination angle of the tibial plateau [20]. Similarly, Lo et al. reported that in patients with KOA, the severity of knee varus deformity was positively correlated with the subchondral BMD of the medial tibia platform [26]. However, these studies can only reflect the results of the change in periarticular BMD caused by the uneven change in mechanical stress, and it is difficult to explain the influence of BMD on the change in limb alignment. Moreover, local BMD could not represent the general bone mass, and it was difficult to reveal the relationship between the general bone mass state and limb alignment.

In this study, we measured the BMD at the lumbar spine, hip joint and femoral neck to represent the state of general bone mass and measured the different angles of limb alignment to evaluate the relationship between knee varus deformity and systemic BMD. We found that the BMD values of the above three sites in varus deformity patients were significantly lower than those in the normal limb alignment group, and the prevalence of osteoporosis was higher than that in the normal limb alignment group. Furthermore, after adjusting for confounding factors, such as age, BMI, and pain duration, regression analysis showed that the BMD at the lumbar spine (L1-L4), the left femoral neck, and the left hip could have a significant positive influence on the value of the HKA, suggesting that a generalized low bone mass status may influence the severity of varus deformity in patients with KOA. Consistent with the results of this study, Im et al. also reported that proximal femur BMD was negatively correlated with KL grading and positively correlated with medial tibiofemoral joint space and the HKA [27].

In recent years, substantial evidence has shown that subchondral bone and cartilage are a closely related integrated unit and mutually adaptable [28–30]. In the early stage of KOA, the absorption of subchondral bone is enhanced, and the mechanical strength of the bone is reduced. Improper stress stimulation also induces subchondral microfracture, which causes bone marrow edema and then degeneration of the cartilage covered on the surface [31, 32].Chen’s study on tibial plateau specimens of patients with knee osteoarthritis found that subchondral bone cysts degeneration was caused by increased local bone remolding, and the structural change in the cysts region accelerated the destruction of the overlying cartilage [33]. Zhu et al. even reported that the source of pain of KOA might be related to the abnormal activity of subchondral osteoclasts, which secreted Netrin-1 and induced neuronal axonal growth [34]. Therefore, we speculated that if patients with KOA also have osteoporosis, their subchondral mechanical properties will be further reduced, which will accelerate the progress of knee osteoarthritis and even varus deformity.

MDFA reflects the bone morphology of the medial femoral condyle, while MPTA reflects the inclination of the medial tibial plateau and JLCA is related to the narrowing degree of the medial articular space. Interestingly, in our study, BMD at the lumbar spine, femoral neck and hip joint is not only significantly correlated with the HKA, but significantly positively correlated with MTPA and negatively correlated with JLCA, indicating that with decreasing BMD, the more severe the inclination of the medial tibial plateau and the narrower the medial articular space would be. In addition, in the normal limb alignment group, the HKA was only negatively correlated with JLCA but not significantly correlated with MDFA and MPTA. In contrast, HKA was not only negatively correlated with JLCA but also positively correlated with MDFA and MPTA angles in the varus deformity group. The above results indicate that varus deformity of the knee is not only related to the narrowing of the medial articular space but may also be related to the change in the bone structure around the joint. Similar studies conducted by Wang’s group have also reached consistent conclusions, pointing out that osteoporosis is an important risk factor for inclination of medial tibial plateau, possibly because osteoporosis exacerbates the severity of subchondral microfractures in patients with knee osteoarthritis.

In fact, subchondral bone provides structural support for articular cartilage and plays a role in shock absorption, and its mechanical structure changes are closely related to cartilage degeneration [35]. Considering the increased fragility of the subchondral bone in osteoporosis patients, subchondral bone remolding will be further enhanced in the early stage of KOA [36]. At the same time, the stress of the medial platform is higher than that of the lateral platform under physiological conditions [37]. Therefore, we hypothesized that when KOA patients have osteoporosis, due to the weakening of the subchondral bone strength and improper stress stimulation, the medial platform may subside, thus aggravating the existing varus deformity caused by cartilage wear. In this study, MTPA was positively correlated with BMD values of the three different sites, which can confirm our inference to a certain extent. With the progression of knee varus, the stress in the medial compartment will further increase, which will aggravate the cartilage damage, thus forming a vicious circle. One study also reported that osteoporosis causes attrition of bone structure in advanced KOA, aggravating the deformity of joints [38]. Therefore, these results suggest that maintaining the systemic bone mass of patients may effectively delay the progression of KOA, especially in patients with osteoporosis.

There are several limitations in this study. First, the biggest limitation of this study is that due to the cross-sectional design, we were only able to determine the association between osteoporosis and varus deformity. Prospective longitudinal studies are needed to determine the causal relationship between the two. Second, as a deficiency of estrogen is a common cause of osteoporosis and KOA, an osteoporosis clinical merger with KOA is often seen in postmenopausal women, so we only included postmenopausal Chinese women. To extend the conclusion to men and other races, research with a large sample of other populations should be conducted to confirm our findings. Third, we failed to observe the changes of BMD in the subchondral bone, as the BMD around the knee joint is not the regular detection area of BMD in our hospital. Therefore, it is necessary to further observe the correlation between periarticular BMD and systemic BMD in patients with KOA in future studies. Despite these limitations, this study still revealed the relationship between the systemic bone mass status and different angles of limb alignment and proposed that osteoporosis may be an independent risk factor for varus deformity. Our study results will also provide a reference to further reveal the internal relationship between osteoporosis and knee osteoarthritis.

## Conclusions

In summary, the incidence of knee varus deformity is not only related to the narrowing of the joint space but also to the change in the bone structure around the joint, which may be an important factor. Osteoporosis should be a major risk factor for varus deformity in postmenopausal women with KOA. The progression of varus deformity of the knee should be concerned in postmenopausal women who simultaneously has KOA and osteoporosis, but more longitudinal studies are needed to confirm this conclusion.

## Data Availability

The data that support the findings of this study are available from the First Affiliated Hospital of Guangzhou University of Chinese Medicine, but restrictions apply to the availability of these data, which were used under license for the current study, and so are not publicly available. Data are however available from the authors upon reasonable request and with permission of Ethics Committee of the First Affiliated Hospital of Guangzhou University of Chinese Medicine.

## Abbreviations

KOA: Knee osteoarthritis
BMD: Bone mineral density
HKA: Hip-knee-ankle angle
MDFA: Medial distal femoral angle
MPTA: Medial proximal tibial angle
JLCA: Angle of the joint line
KL: Kellgren-Lawrence score
BMI: Body mass index
WHO: World Health Organization
